# Lessons from the field: adapting lymphatic filariasis morbidity mapping and patient care amidst conflict in Ethiopia

**DOI:** 10.1093/inthealth/ihag035

**Published:** 2026-05-08

**Authors:** Fikre Hailekiros, Mebratu Mitiku, Merga Mekonnon, Tesfahun Bishaw, Adisu Abebe, Louise A Kelly-Hope

**Affiliations:** National Podoconiosis Action Network (NaPAN), P.O. Box 2255/1110, Addis Ababa, Ethiopia; National Podoconiosis Action Network (NaPAN), P.O. Box 2255/1110, Addis Ababa, Ethiopia; Ministry of Health Ethiopia, Neglected Tropical Diseases Desk, P.O. Box 1234, Addis Ababa, Ethiopia; Ministry of Health Ethiopia, Neglected Tropical Diseases Desk, P.O. Box 1234, Addis Ababa, Ethiopia; Amhara Regional Health Bureau, Neglected Tropical Disease desk, P.O. Box 495, Bahir-Dar, Ethiopia; Institute of Infection, Veterinary and Ecological Sciences, University of Liverpool, L3 5RF, Liverpool, UK

## Abstract

Conflict in Ethiopia has prevailed in recent years and is impeding the implementation of lymphatic filariasis elimination activities for morbidity management and disability prevention. This led to the need for alternative approaches to ensure safe and effective implementation. Here, we outline the challenges that were faced (e.g., insecurity, restricted travel to external field teams, limited in-person training, direct patient care). First, we describe what potential solutions were discussed and decided. Second, we summarise the planned adapted strategy and how it was implemented through central-level planning, supervision, courier-based data transfer, and centralised data management. Third, we highlight what worked well, what did not, and key lessons learned. Finally, we outline areas for future consideration, including for example, the importance of global and national standard guidelines, and a better understanding of type and intensity of the conflict to help with realistic and safe planning.

## Background

Lymphatic filariasis (LF) is a neglected tropical disease (NTD) that causes chronic disability through hydrocoele and lymphoedema. These conditions not only reduce productivity but also impose stigma and social exclusion.^1,2^ LF morbidity, particularly lymphoedema and hydrocoele, imposes significant socioeconomic burdens in Ethiopia, including reduced productivity, income loss, caregiver strain and social exclusion.^3,4^ Ethiopia has committed to eliminating LF as a public health problem in line with WHO global targets. Morbidity mapping is critical for identifying patients, planning care and thereby ensuring equitable and sustainable access to Morbidity Management and Disability Prevention services.^5^

The National Podoconiosis Action Network (NaPAN), Addis Ababa, Ethiopia, supports the National NTD programme with selected LF activities. Under routine circumstances, LF morbidity mapping is conducted through door-to-door visits by Health Extension Workers (HEWs). Training is provided in target woredas (local administrative areas), with HEWs serving as data collectors, Health Center Heads as supervisors and District Health Office Heads as team leaders. Completed questionnaires are collected in person by the field team, checked for consistency and validated before data entry. This system ensures high-quality data, local ownership and accountability, while also linking patients to care services.

## The challenges

**Figure 1.**
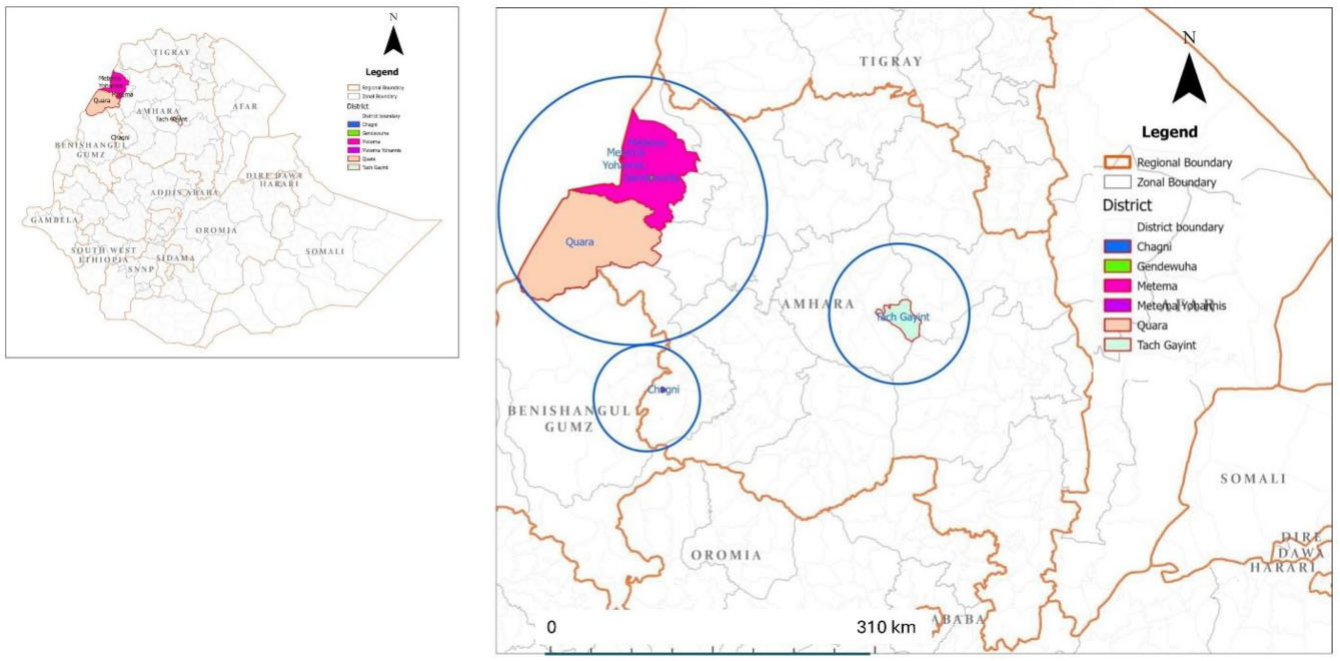
Ethiopian Study Areas.

The morbidity mapping exercise was conducted during July-December 2024 in six districts of the Awi, South Gondar and West Gondar zones of Amhara Regional State (Fig.1). None of the study districts included in this survey are co-endemic for LF and podoconiosis, although LF–podoconiosis co-endemic areas are present in other parts of Ethiopia. Insecurity posed major obstacles in the study districts, including the following:

- Ongoing insecurity and conflict made travel to target woredas unsafe for the NaPAN staff and other regional staff from outside the local area.
- Direct training and supervision of HEWs was not feasible.
- Exposing external healthcare workers to conflict zones could disrupt services and endanger lives, while patients risked being left unidentified and unsupported.
- Without adaptation, both morbidity mapping and patient care services risked being delayed or abandoned, undermining Ethiopia’s LF-elimination roadmap.

## Potential solutions and planning

In response to the identified challenges, the NaPAN team considered several options including the following, and evaluated their advantages and disadvantages.

- **Postponement**: delaying mapping and care until security improved, although this risked losing momentum and leaving patients without support.
- **Relocation to safer areas**: moving the project to the southern part of the country, where security conditions were more stable. While feasible, this option would have excluded patients in Amhara from immediate care and undermined equity.
- **Remote training**: using digital platforms, although connectivity and access were limited in rural Ethiopia.
- **Centralized approach**: bringing district leaders to a safe location for training and equipping them to cascade responsibilities to HEWs in the affected areas.

Ultimately, the centralized approach was selected as the most feasible, balancing safety and continuity while ensuring that patients in conflict-affected areas were not excluded from care. See Table 1 for a summary of the standard vs conflict-adapted LF morbidity mapping approaches.

**Table 1.** Comparison of standard vs conflict-adapted LF morbidity mapping approaches.

Step	Standard practice	Adapted practice (conflict setting)
Training	In target districts, the Data Collectors (i.e. HEWs), Supervisors (Health Center Heads) and Team Leaders (District NTD Programme coordinators) are trained by NaPAN staff	In the regional capital (Bahir-Dar), Health Center Heads and District Health Office Leaders were trained by NaPAN staff and then the Head and Leaders cascaded these to local supervisors and data collectors in their respective districts
Distribution of data collection tools	In target districts, the printed questionnaires are directly provided to Data Collectors after the training by the NaPAN team	During the centralized training in Bahir-Dar, the printed questionnaires were given to the Heads and Leaders so that they could distribute them directly to local Data Collectors in respective districts
Data collection method	By trained HEWs through door-to-door visits	No modification
Supervision and data quality check	Data quality is checked by district Supervisors and Team Leaders and by NaPAN staff during questionnaire data collection	Data quality was checked by Supervisors in respective catchment areas and ensured completeness and consistency. The NaPAN staff made phone calls after the questionnaires reached Addis Ababa and crossed-checked the data during the entry process to validate it
Data transfer and payments	Completed questionnaires are collected and transferred by NaPAN staff in person from study districts and effect payments for data collectors	Completed questionnaires were sent by courier services to Addis Ababa and payments were made by bank transfer

## Implementation of a centralized approach

*How it went:*

- **Centralized training and tool distribution**: Health Center Heads and District Health Office Leaders convened in the regional capital, Bahir-Dar. The NaPAN team provided training and printed questionnaire data collection tools for the leaders so that they could be distributed to the local data collectors in their respective catchment areas.
- **Delegated responsibility**: the trained leaders then cascaded training, data collection tools and responsibilities to the local data collectors (i.e. HEWs) in their own districts, who conducted door-to-door case identification and patient registration.
- **Supervision**: data quality was verified by supervisors in each catchment area, with additional phone-based checks by the NaPAN team.
- **Courier-based data transfer**: completed forms detailing morbidity mapping data were sent to Addis Ababa via courier services for data entry.
- **Centralized data entry and validation**: validation and entry were conducted at the national level, ensuring that questionnaires were completed and consistent.

*What worked:*

- Mapping was completed across six districts despite insecurity. The morbidity mapping exercise identified a total of 1121 patients across the six study districts. Table 2 summarizes the distribution of lymphoedema, hydrocoele (18.5%), combined cases and other forms of lymphoedema by district, highlighting the high burden of leg lymphoedema (n=713/1121; 63.6%), which represents around two-thirds of all cases. Of the districts, Tach Gayint in South Gandar reported 602 cases, approximately one-half (53.7%) of the 1121 cases found.
- Hydrocoele and lymphoedema cases were successfully identified and registered.
- Patients were connected to local care services through existing health structures.
- The safety of all healthcare workers was prioritized and they did not come to harm.
- Data quality was safeguarded through centralized validation.

**Table 2.** Number of LF morbidity cases identified by district in the Amhara region, Ethiopia.

**Zone**	**District**	**Lymphoedema**	**Hydrocoele**	**Both lymphoedema and hydrocoele**	**Other (breast and arm) lymphoedema**	**Total (% of total)**
Awi	Chagni	179	13	0	11	203 (18.1%)
South Gondar	Tach Gayint	394	95	21	92	602 (53.7%)
West Gondar	Gendawuha	10	3	1	8	22 (2.0%)
	Metema	75	71	5	34	185 (16.5%)
	Metema Yohannis	3	3	0	0	6 (0.5%)
	Quara	52	22	5	24	103 (9.2%)
Subtotal (% of total)		713 (63.6%)	207 (18.5%)	32 (2.9%)	169 (15.1%)	1121

*What did not work:*

- The lack of direct supervision by and presence of NaPAN staff reduced the opportunities for immediate troubleshooting.
- Delays occurred in data transfer due to courier logistics, preventing real-time analysis.
- There were limited opportunities for on-the-ground mentoring of HEWs and understanding of the patient burden.

## Key lessons

This adapted approach led to three main lessons being learned. First, we found that centralized training and remote supervision proved effective in implementing the morbidity mapping when direct field access was restricted. Adapting established protocols to the realities of insecurity ensured continuity without compromising safety. Second, it was vital to harness local leadership, including the district Health Office Heads and Health Center Leaders, to play a crucial role in bridging operational gaps. Their involvement reinforced accountability, fostered ownership and maintained momentum in the absence of direct external supervision. Finally, we found that leveraging existing courier services to transport the completed survey forms reduced the risks faced by members of the external NaPAN team. These approaches reduced delays, safeguarded data quality and minimized the risks associated with physical transport in insecure areas.

## Future development

The insecure situation in Ethiopia is an ongoing challenge and more defined strategies for tackling these complex situations are urgently needed. Currently, there are no standard global or national guidelines on insecurity for NTD programmes,^2,6^ and a better understanding of the type and intensity of the conflict may help with planning and assessing the level of security required. Open access, geo-referenced conflict data are available from the Armed Conflict Location & Event Data (ACLED) portal and can be better used in the future. The data available for 2024 clearly show the high-risk areas and key actors involved.^7^ The NaPAN team also aims to explore the use of digital tools for remote training, supervision, patient follow-up where connectivity allows, as well as the facilitation of data entry at district level by trained encoders with access to computers. Finally, it is important to strengthen documentation and the sharing of lessons learned, along with robust contingency planning in fragile settings, including relocation strategies or flexible implementation options when necessary.

## Data Availability

The data underlying this article are maintained at the National Podoconiosis Action Network (NaPAN). Both the original questionnaires and the electronic soft copy are securely stored and available upon reasonable request to the corresponding author.
